# Nanomaterials and Nanotechnology-Associated Innovations against Viral Infections with a Focus on Coronaviruses

**DOI:** 10.3390/nano10061072

**Published:** 2020-05-31

**Authors:** Mahmoud Nasrollahzadeh, Mohaddeseh Sajjadi, Ghazaleh Jamalipour Soufi, Siavash Iravani, Rajender S. Varma

**Affiliations:** 1 Department of Chemistry, Faculty of Science, University of Qom, Qom 37185-359, Iran; mhd.sajjadi@gmail.com; 2 Radiology Department, School of Medicine, Isfahan University of Medical Sciences, Isfahan 81746 73461, Iran; Jamalipour@med.mui.ac.ir; 3 Faculty of Pharmacy and Pharmaceutical Sciences, Isfahan University of Medical Sciences, Isfahan 81746 73461, Iran; 4 Regional Centre of Advanced Technologies and Materials, Palacký University in Olomouc, Šlechtitelů 27, 783 71, CZ-779 00 Olomouc, Czech Republic

**Keywords:** nanotechnology, nanoparticles, quantum dots, graphene oxide, nanovaccines, coronaviruses, COVID-19, viral infections, SARS-CoV-2

## Abstract

Viral infections have recently emerged not only as a health threat to people but rapidly became the cause of universal fatality on a large scale. Nanomaterials comprising functionalized nanoparticles (NPs) and quantum dots and nanotechnology-associated innovative detection methods, vaccine design, and nanodrug production have shown immense promise for interfacing with pathogenic viruses and restricting their entrance into cells. These viruses have been scrutinized using rapid diagnostic detection and therapeutic interventional options against the caused infections including vaccine development for prevention and control. Coronaviruses, namely SARS-CoV, MERS-CoV, and SARS-CoV-2, have endangered human life, and the COVID-19 (caused by SARS-CoV-2) outbreak has become a perilous challenge to public health globally with huge accompanying morbidity rates. Thus, it is imperative to expedite the drug and vaccine development efforts that would help mitigate this pandemic. In this regard, smart and innovative nano-based technologies and approaches encompassing applications of green nanomedicine, bio-inspired methods, multifunctional bioengineered nanomaterials, and biomimetic drug delivery systems/carriers can help resolve the critical issues regarding detection, prevention, and treatment of viral infections. This perspective review expounds recent nanoscience advancements for the detection and treatment of viral infections with focus on coronaviruses and encompasses nano-based formulations and delivery platforms, nanovaccines, and promising methods for clinical diagnosis, especially regarding SARS-CoV-2.

## 1. Introduction

Presently, the outbreak of viral respiratory infections is rampant and spreading all over the world, and researchers are urgently aiming to identify and develop suitable nanovaccines and therapeutic options including emerging nano-based options. Viruses are a small obligate intracellular parasite and are non-living outside a host cell, whose interactions with them mostly include receptor-ligand interactions; respiratory viruses, among them, represent serious threats for all demographics. Importantly, the need for drug design and discovery is prompted by inherent characteristics of viral illnesses that contain complexities in lifecycle stages, main alterations in replication dynamics, diverse stages of replication in assorted subcellular organelles or compartments, development of drug resistance, and the feasibility of latent viral infections in an inaccessible biological compartment, among others. The quick advancement of drug resistance to recently accessible treatments and the adverse health impacts owing to their protracted use has become a critical health issue. Over the last 20 years, few old illnesses have re-emerged, and many new infectious disease agents, especially viruses, have appeared, with human immunodeficiency virus (HIV) and acute respiratory infections being the leading causes of death worldwide [1,2,3,4]. Owing to emergence of a diversity of novel variants of pathogenic viruses, the lack of immunization and therapy of infectious illnesses have become a critical challenge and a clinical threat in an exceedingly short span of time.

**Figure 1:**
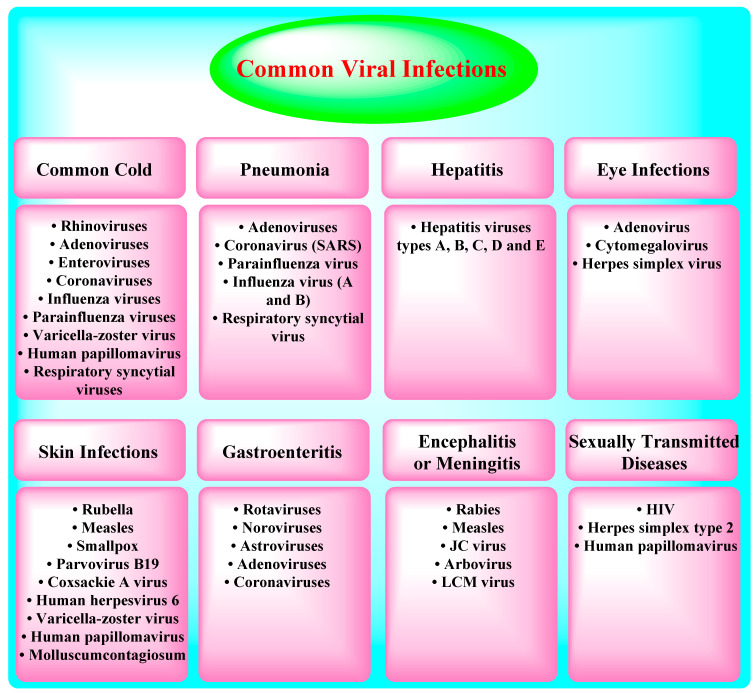
Types of common viral infections, the details are obtained from Ref. [9].

**Figure 2:**
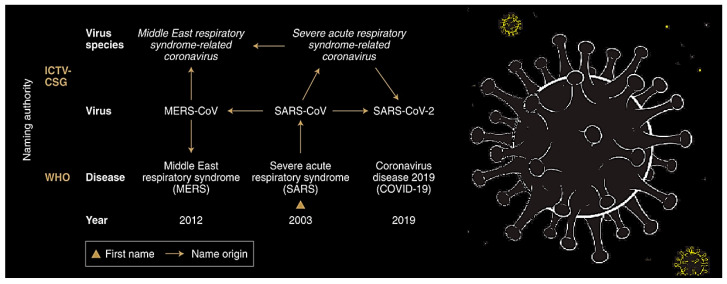
SARS-CoV, MERS-CoV, and SARS-CoV-2 classification. Redrawn from [12], an open access article (CC BY).

The human respiratory mucosa is the most significant portal and the primary site of entry for incursion or infection by various viruses such as the influenza virus, the respiratory syncytial virus, and the parainfluenza virus, among others. These virulent pathogens initially infect the upper respiratory tract and then reach the lower breathing airways (or lower respiratory tract), leading to death. Lower respiratory tract illnesses explicate a leading cause of human morbidity and mortality with ~3 million deaths annually worldwide [5]. These pathogens often enter the host via airborne transmissions (animal/human-to-human) and then spread through direct contact or droplets/aerosols, effectively replicate in the respiratory tract, and mostly cause clinical symptoms/manifestations, namely fever, dyspnea, cough, bronchiolitis, and/or pneumonia [6]. Most respiratory tract infections are due to one of the numerous respiratory viruses, mostly influenza virus, respiratory syncytial virus (RSV), rhinovirus (RV), and severe/acute respiratory syndrome (SARS), infecting the person’s lower respiratory tract [7,8,9,10]. Nevertheless, adenovirus, parainfluenza virus, enterovirus, and human bocavirus (HBoV) and human metapneumovirus (HMPV) can also play relevant roles, especially during epidemics [11]. Most of these infections (~80%) are caused by the viruses summarized in Figure 1 and Figure 2 [9].

Respiratory viruses generally present a main health issue in infants, children, elderly people, and/or immunocompromised patients. Parainfluenza virus [13] and/or RSV [14] infections are the leading causes of hospitalization for acute respiratory illnesses in children, leading to 40% and 45% of pediatric hospitalizations, respectively. Among these viral infections, 3~5% of the lower respiratory tract illness cases in children are caused by adenovirus infections [15]. SARS, the most recent infectious illness, is generally an acute form of bronchopneumonia, which could be caused by a novel coronavirus (CoV). Astoundingly, CoVs are the main pathogens that primarily target the human respiratory system. The first emergence of “SARS-CoV” infections (8-month outbreak) had a fatality rate of ~10%, which resulted in over 8000 confirmed cases [10]. In 2012, after ten years, the Middle East respiratory syndrome “(MERS)-CoV” emerged in a universal outbreak, which was transmitted to almost 27 countries (https://www.who.int/emergencies/mers-cov/en/; 2013). At the end of 2019, 2019-nCoV (emerging novel CoV), as a potentially deadly “SARS-CoV-2” (severe/acute respiratory syndrome CoV 2) presented a critical threat and a global concern to vulnerable populations within an extremely short time span [16]. Astoundingly, these emerging SARS-CoVs are more likely to ensue in the elderly population, particularly those who suffer from hypertension, diabetes, and cardiovascular or coronary heart illness. At least half of these patients are tremendously susceptible to infection, which may turn into a cytokine storm, septic shock, metabolic acidosis coagulation dysfunction, acute respiratory distress syndrome (ARDS), heading to demise. We are today witnessing a global transmission of SARS-CoV-2 with more than 4,178,156 positive cases and at least over 286,353 deaths, transmitted from China to over 212 countries/regions. Now, various antiviral agents and proposed drugs are under clinical trial evaluations for their potential effects against SARS-CoV-2, but they are in the initial stages, and crucial issues, including pharmacokinetic characteristics, drugs availability, and potential side effects, should be analyzed astutely [17].

Efficient treatments against viruses are hindered by the advancement of drug resistance and non-specific targeting, particularly those associated with influenza [18] and HIV [19,20]. Indeed, the main challenges to combat such epidemic viral infections are that for several of them (especially SARS-CoV infections) there are no effective antiviral drugs or specific treatments accessible, thus designing potent drugs to combat these diseases needs to be pursued urgently. Collectively, control or prevention of the pathogenicity of viruses is probably the best and possibly only way to diminish public health challenges resulting from viral illnesses. Efforts to control these respiratory infections are focused on prevention via vaccine advancement.

This perspective begins with a broader overview of newer strategies in vaccine design with illustration of the nano-based formulation approaches against viruses that are being developed as delivery vehicles, including novel nanovaccine transfer platforms and useful nanodrugs for treatment of viral infection.

## 2. Nanotechnology against Viral Infections with Focus on CoVs

**Figure 3:**
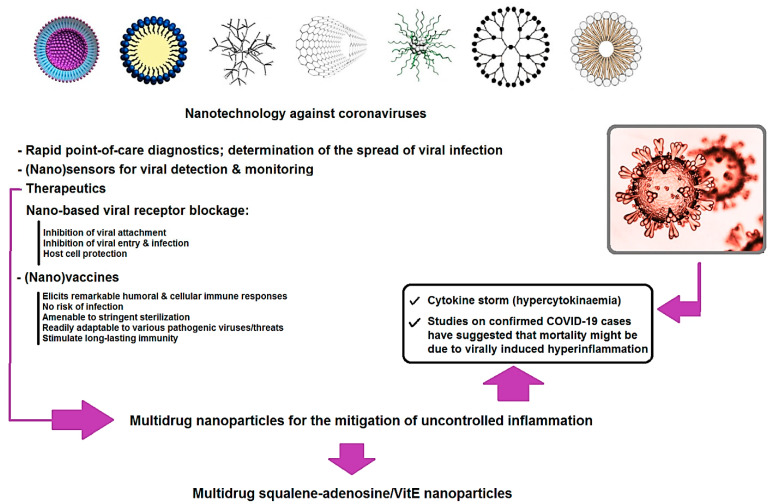
Nano-based approaches against viruses, especially coronaviruses (CoVs).

Nanotechnology and nanoscience refers to the improvement, advancement or application of atomic/molecular structures with at least one dimension that is in the nanoscale range (1~100 nm) with variable composition, shape/morphology, size, or surface properties [21,22,23,24]. Over the decades, NPs/nanocarriers have been utilized in a diversity of new pharmaceutical applications, namely effective delivery of drugs to the targeted sites without exposing the healthy tissue cells, sensitive imaging to detect viral illnesses at early stages, and crossing these barriers (e.g., the epithelial/endothelial, immunological, or cellular barriers) to deliver nano-therapeutic molecules or nanovaccines to specific diseased organs or cell tissues as well as to interact with biomolecules in the blood or within organ tissues. Additionally, the inactivation of viruses by using engineered nanomaterials (acid functionalized multi-walled carbon nanotube comprising photo-activated molecules) and inhibition of viral binding with the host cell surface receptor are important examples of nano-based approaches against viruses (e.g., angiotensin-converting enzyme 2 (ACE2) receptor, especially in the case of SARS-CoV-2) (Figure 3). Besides their nanoscale size, nanocarriers can efficiently deliver antigens owing to ease of surface functionalization and have capability to co-transport antigens accompanied by numerous adjuvants. One of the significant requirements for any efficient (nano)medicinal agent is the delivery to the proper place at the appropriate concentrations within a suitable time period [25].

Two kinds of passive or active targeting mechanisms are commonly possible. In a targeted nanoparticulate delivery system, passive targeting could occur because of the enhanced permeability or leakiness (causing by inflammation or malignancy) of a local vasculature, which can result in the sick section growing more accommodating to the buildup of nanotherapeutic agent. Instead, active targeting is associated with attachment of targeting ligands (e.g., antibodies, carbohydrates, peptides, proteins, etc.) to direct the nanotherapeutic entity to a specific receptor, site, or epitope. Incorporation of the nuclear localization signals on the nanocarrier in active targeted nanoparticulate delivery systems is thus desirable to enhance specificity. Active targeting is thus a significant requirement for the remediation of virus infections since numerous antiviral nanodrugs are essential to ensure they are confined at particular subcellular organelles/regions, depending on the replication stages and the (nano)drug’s modes of action. Along this line, integrase inhibitors effectively prevent the strand transfer reaction of the virus lifecycle stages (e.g., HIV integrase, which is responsible for the attachment of viral DNA into host/cell chromosomes) [26].

Other unmet enquiries in viral management options comprise the utilization of RNA-mediated interference technology, which is a promising molecular tactic for the handling of a variety of infectious illnesses [27]. Indeed, there are limitations that prohibit validity and clinical utility of small/short interfering RNA (siRNA) therapeutics, including the inability of RNA to traverse the membrane of a cell (owing to the big molecular weight and/or negative charge), as well as expeditious renal sanction, uptake, or poisonousness (owing to aroused immune reaction) [27,28]. To overcome such restrictions, the incorporation of siRNA within or onto the NPs/nanocarriers could attain successful viral replication inhibition; developments of efficient and novel treatment approaches are thus imperative. For instance, silver NPs with antiviral effects were green synthesized using curcumin as a reducing agent; curcumin modified silver NPs had a remarkable inhibitory effect against respiratory syncytial virus (RSV) infection [29]. Nanotechnology-based platforms for nanotherapeutic drugs that are successfully approved or under further investigation for treating or inhibiting viral infections are summarized in Table 1. Additionally, some significant engineered nanomaterials for the diagnosis and inhibition of CoVs are summarized in Table 2.

The present medications for MERS-CoV are engendered from H1N1 flu and SARS-CoV outbursts [54,55,56,57]. These incorporate various mixes of little particles with expansive antiviral action (e.g., corticosteroids, interferons, and ribavirin) and polyclonal/monoclonal antibodies for therapeutic options [56,58]. Membrane-anchored glycoprotein S has of late been seen as a basis for the connection between the host cell and MERS-CoV [58,59], and the improvement of MERS-CoV entrance inhibitors focusing on the S1 subunit can be deemed as a suitable antiviral practice. As of late, nanomaterials have attained favorable stature to alter the cycle of viral infection [60]. The virus entrance into the host cells might be supported by multivalent connections. The similar trait of nanostructured materials possessing high surface to volume proportion and permitting the addition of ligands on their surfaces may thus meddle with viral connection and block its entrance into cells [57].

In one investigation, heptad repeat 1 (HR1) peptide inhibitors were examined for inhibiting HR1/HR2-mediated sheath merging among MERS-CoV and host cells, the main conduit for host infections induced by MERS-CoV [61]. Mainly, the inhibitory influences of peptide pregnancy-prompted hypertension have been accelerated 10-fold by generation of a gold nanorod complex. Additionally, this complex showed improved biocompatibility and metabolic strength, both in vivo and in vitro, thus successfully forestalling sheath merging prompted by MERS-CoV [61].

Additionally, triangular star-shaped bovine serum albumin (BSA)-coated tellurium NPs reportedly suppressed virus infection predominantly by obstructing the virus internalization procedure; these nanostars showed remarkable antiviral activities against porcine epidemic diarrhea virus (PEDV) (e.g., CoV) [62].

### 2.1. Nano-Based Advances for Viral Detection

With the evident possibility of enormous outbreaks, the situation is very quickly growing more critical, and the need for the development of quick diagnostics and effective control strategies is becoming graver. The diagnostic field was boosted early in the 1980s with two main advancements, namely the birth of diverse immunoassays and the invention of polymerase chain reaction (PCR). The first study on the serological detection technique of viruses with different immunoassays was described in 1970, in which a radioimmunoassay was utilized towards the detection of the Australia antigen, which was later labeled, the HBs Ag (hepatitis B surface antigen) [63]. In the mid-20th century, electron microscopy and cell/tissue culture techniques were discovered that are still usually employed for the direct detection of diverse viruses [64] and subsequently led to an array of serological and molecular detection techniques. Molecular techniques are comparatively more sensitive and are quicker than immunoassays and could be utilized not only in a facile form towards the manual detection of a virus but also as the embedded component of more complex systems. Despite these promising applications, most molecular means still have diverse potential limitations in accuracy, repeatability, specificity, and sensitivity, often caused by the high genetic variability of several viruses. In addition, the traditional techniques for respiratory virus detection are generally costly, time consuming, labor intensive, and require specialized laboratory equipment and expertise.

Nanotechnologies are presently focused on pathogenic viruses and are geared up for detection of viral infections. In this respect, gold (Au) NPs and quantum dots (QDs) have become key components in the improvement/advancement of novel nanotechnology-based detection for several respiratory viruses; Au NPs have been combined with silver staining and utilized for the detection of HPV (human papillomavirus) in a cervical carcinoma cell [65]. Currently, there is an array of nanostructures, namely metal NPs, graphene oxide (GO), QDs, silica NPs, polymeric NPs, and carbon nanotubes, that are being seriously scrutinized for virus testing and detection [66,67,68]. The advancements in (nano)biohybrid structures are the common methods for exploiting NPs in virus detection, which can include biomolecules (one or more) derived from viruses. Antibody (Ab), DNA, RNA, antigen, etc. are conjugated on the surface of a diversity of NP forms which enable rapid, sensitive, direct, and facile detection with unprecedented multiplexing capabilities. The surface of Au NPs can effectively serve as soft metal ions, which simply binds to a soft ligand, e.g., thiols. The novel Au NP-based detection approaches have been developed for diverse types of clinically relevant viruses with a particular focus on the Au NP biohybrid systems, virus detection targets, and assay modalities. Table 3 depicts diverse case studies employing several scanometric, electrochemical, fluorometric, or colorimetric systems in Au NP-based detection of viruses [69].

#### Detection of SARS-CoV-2: Innovative Nano-Based Discoveries

The reverse transcription polymerase chain reaction (RT-PCR) has been generally deployed as a routine diagnosis method for the detection of CoVs [70]. However, some false-positive or false-negative reports have been observed, especially in the case of COVID-19. Interestingly, in one study, a double-operational plasmonic biosensor merging the localized surface plasmon resonance (LSPR) sensing transduction and the plasmonic photothermal (PPT) influence offered a promising substitute for the diagnosis of COVID-19, clinically. Delicate recognition of the particular arrangements of SARS-CoV-2 via nucleic acid hybridization has been accomplished using 2-D gold nano-islands decorated with complementary DNA receptors. The thermos-plasmonic heat was produced on the chip for improved sensing performance, whereby illumination at their plasmonic resonance frequency and the ensuing local PPT heat improved the in situ hybridization temperature and enabled the precise discernment of two similar gene sequences. This biosensor showed remarkable sensitivity for the examined SARS-CoV-2 sequences with a much lowered detection limit of 0.22 pM concentration and permitted specific target recognition in a multigene mixture [70]. Qui et al. [70] reported that by applying LSPR excited at two various wavelengths and the plasmonic resonances of PPT, remarkable stability, reliability, and sensitivity in diagnosis are achievable.

**Figure 4:**
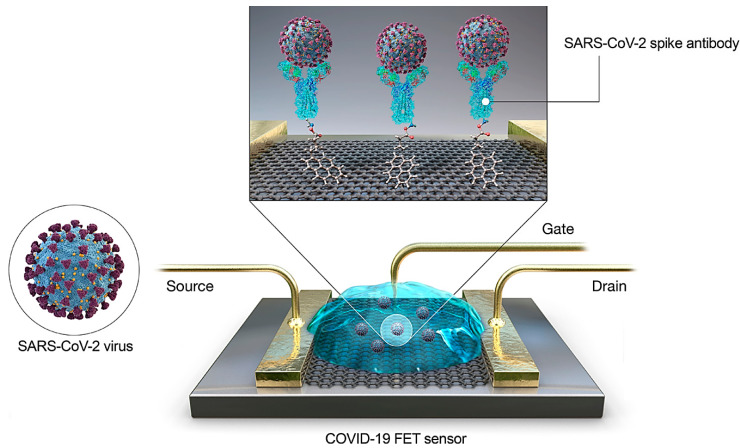
Field-effect transistor (FET) sensor and its related operational process for the diagnosis of COVID-19. Reproduced (adapted) with permission from [71]. Copyright ^© 2020^, American Chemical Society.

The detection of SARS-CoV-2 in medical samples could be accomplished by a field-effect transistor (FET)-based biosensing gadget, and its performance and efficacy was evaluated using cultured virus, antigen protein, and nasopharyngeal swab examples from COVID-19 patients (Figure 4) [71]. For fabrication of the biosensor, the graphene sheets of the FET were coated with precise Ab against SARS-CoV-2 spike protein; the graphene sheets were decorated with the SARS-CoV-2 spike antibody through 1-pyrenebutyric acid *N*-hydroxysuccinimide ester as a probe linker. Accordingly, the produced FET tool could identify the SARS-CoV-2 spike protein at concentrations of 1 fg mL^−1^ in phosphate-buffered saline and 100 fg mL^−1^ medical transfer vehicle. Additionally, this sensor effectively identified SARS-CoV-2 in culture (with limit of detection ~1.6 × 101 pfu mL^−1^) and medical tests (with limit of detection ~2.42 × 102 copies mL^−1^). The prepared FET biosensor showed good sensitivity for diagnosis of COVID-19 with no sample pretreatment or labeling, but various other materials could be investigated to improve the signal-to-noise ratio [71].

### 2.2. Nanotechnology for Designing Vaccines

**Figure 5:**
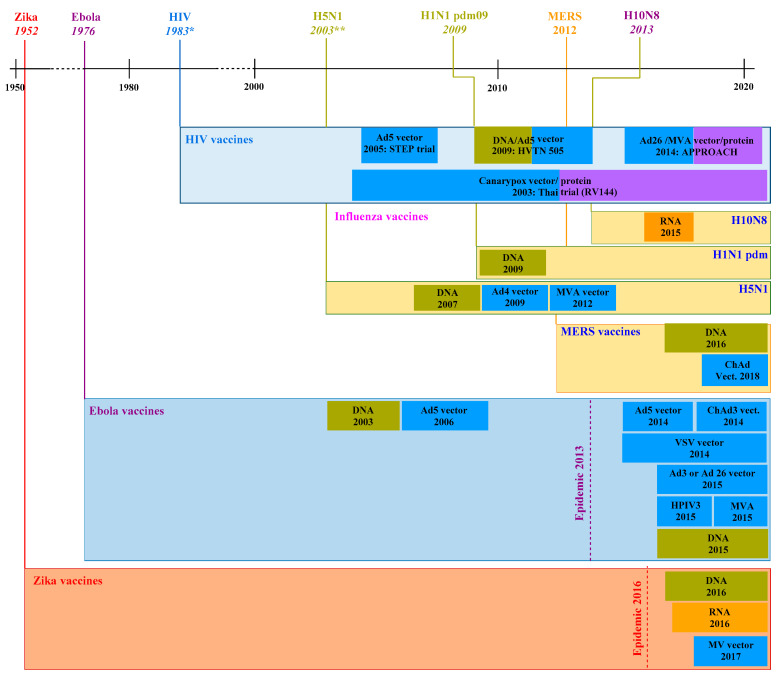
The onset of the clinical vaccine’s development against massive outbreaks. Ad4, 5, or 26 vectors (human adenovirus type 4, 5, or 26); ChAd (chimpanzee adenovirus); MVA (modified vaccinia Ankara); VSV (vesicular stomatitis virus); HPIV3 (human parainfluenza virus type 3); MV (measles virus). Redrawn with permission from [72], an open access article (CC BY).

**Figure 6:**
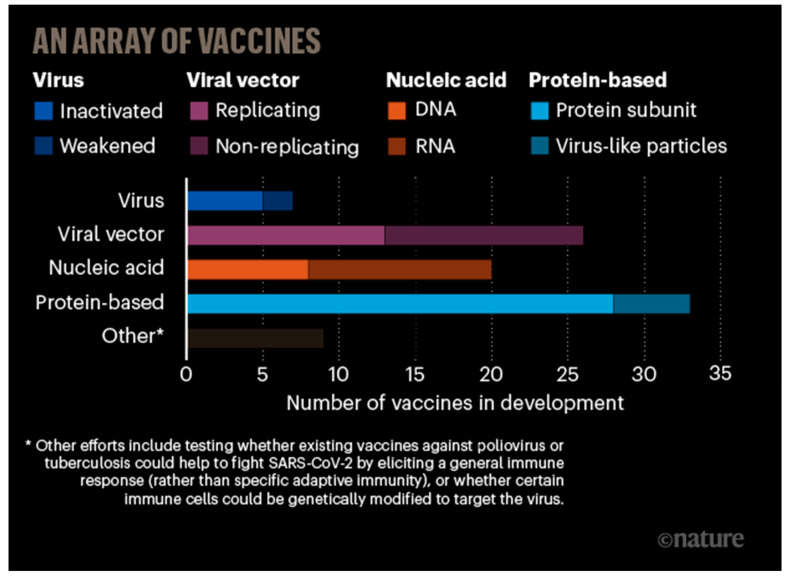
Eight types of explored vaccines against CoV, based on various viruses or viral parts. Redrawn from [73]. The details are obtained from WHO COVID-19 Vaccine Landscape and [74,75,76].

The disruptive outbreaks of fatal epidemics and respiratory infectious illnesses have greatly uplifted the progress of modern and effectual vaccine formulations to the status of a worldwide healthcare issue. It is generally known that prevention is invariably better than cure for the control of illnesses. Vaccination is still one of the most extraordinary tactics for preventing, controlling, or ameliorating infectious diseases, as well as administrating the antigenic materials to motivate an individual’s immune systems to develop adaptive and protective immunity against a specific pathogen, especially viruses. To date, vaccination effectiveness has been extensively studied, and it has been authenticated that vaccines are responsible for the impressive control and even elimination of numerous potentially fatal illnesses, such as polio, smallpox, measles, hepatitis A, papilloma, etc. Despite numerous efforts, many other illnesses are still lacking therapeutic or effective prophylactic vaccines towards numerous clinically relevant viruses, e.g., HIV, Zika viruses, hepatitis C viruses (HCVs), influenza virus (e.g., H5N1, H1N1, etc.), cytomegaloviruses (CMV), Ebola viruses, RSV, and SARS-CoVs [64]. Figure 5 and Figure 6 present the general timelines for the vaccine development against viral infections [72], including the year when given viruses started transmitting in the human population, the start of clinical development of vaccines, and the employed technology that has been shown to work particularly well for a viral vector or nucleic acid-based vaccines. For HIV, in 1983 (the discovered year), only selected investigations that may be considered a major advance are shown. Similarly, 2003 illustrates the year H5N1 triggered several contagions, although H5N1 in humans was first registered in 1997.

**Figure 7:**
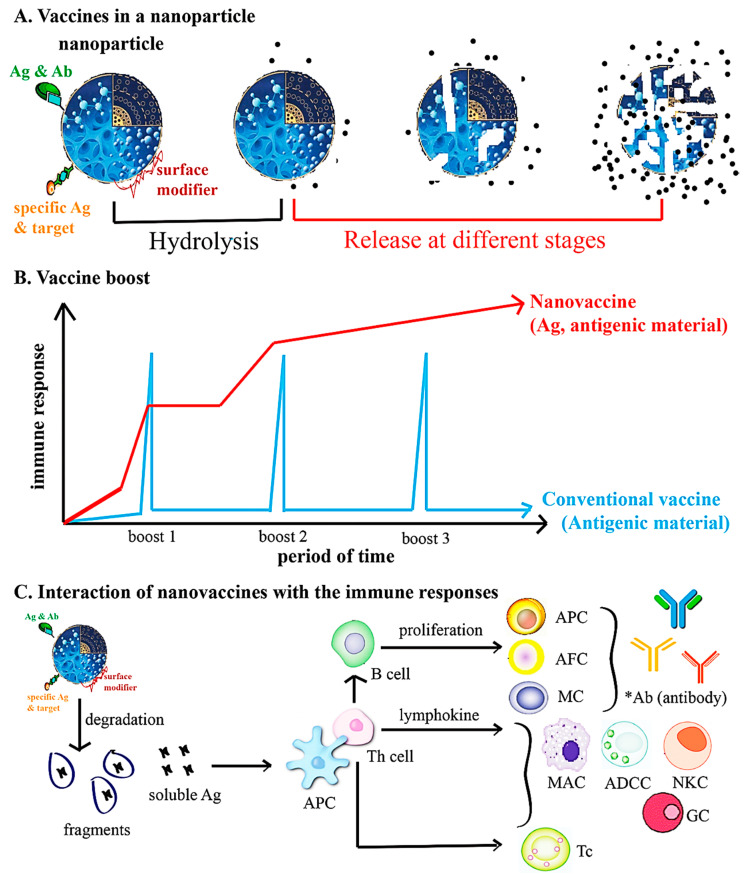
Nanotechnologies utilized in nanomedicine vaccines. Ag (antigen); Ab (antibody); APC (antigen presenting cell); IgG (serum immunoglobulin G); AFC (antibody forming cell); ADCC (antibody-dependent cytotoxic cell); Tc (cytotoxic T-cell); GC (granulocyte); Th (helper T-cell); MAC (macrophage); MC (memory cell); NKC (natural killer cell). Reproduced with permission from [24], (CC BY).

The conventional vaccines have been produced utilizing inactivated/killed or live-attenuated organisms (first generation), whereas subunit and RNA/DNA vaccines (second and third generations, respectively) are applied to elicit protective immunity against contagious diseases [77,78,79,80,81]. Although subunit and DNA/RNA vaccines have several benefits, namely higher care profile, cost effectiveness, and capability to elicit an immune response against a particular pathogen over conventional vaccines, they suffer from challenges associated with the relatively weak immunogenicity, toxicity, intrinsic instability in vivo, and the requirement for multiple administration. These include the premature degradation of a molecule and the lack of ability to translate into an immunogen (e.g., proteins/peptides, lipids). Thus, there is an increasing need to develop a novel generation of vaccine molecules that can act as a functional immunogen and an adjuvant. Indeed, novel antigen preparations, new strategies for vaccination, as well as consecutive programs of general immunization have recently contributed to improvements in the efficacy of viral vaccines, amongst which the emerging nanotechnology holds enormous promise [82,83]. Furthermore, the frequency of boost, the administration routes, and refrigeration of the vaccines are still challenges for vaccine distribution in smaller and remote villages in some countries. To overcome such hurdles of subunit and conventional vaccines, nanotechnology-based formulations, NP-based vaccine candidates, and NP-based delivery systems with numerous advantages have been recently incorporated into vaccine development (Figure 7 and Table 4) [24].

A large number of nanostructured carriers, namely non-metal NPs, QDs, carbon nanomaterials, polymeric NPs (chitosan, PLGA (poly-lactide-*co*-glycolides), γ-PGA (poly-γ-glutamic acid), metal/metal oxide NPs, silica NPs, carbon black NPs, liposomes, dendrimers, solid lipid nanocarriers, and VLPs (virus like particles) have been intensively designed and investigated to carry a variety of molecules, e.g., drugs, proteins/peptides, DNA/RNA, antibodies, and vaccines for their utility in both antigen delivery (antigen nanocarriers) and as adjuvants to immune cells via an effective attempt to promote a protective humoral immune response. NPs and/or nanocarriers have been vastly studied as vaccine adjuvants since they possess structural and chemical features that increase their immunogenicity; synthesized NPs have illustrated their potential as nanovaccine delivery platforms (Table 5). In this respect, VLPs provide repetitive immunodominant viral epitopes to improve the activation of a particular, robust immune response comprising humoral or cellular immunities [88,89]; the first licensed VLP nanovaccine for human utility was the HBsAg (hepatitis B surface antigen). Nevertheless, the protective efficacy of nanocarrier/NPs against viruses that exploit diverse infection routes are required to be improved and better characterized. It is not clear whether a nasal or oral nanovaccine would achieve a better, long-lasting mucosal immunity or longer-term protection against a particular virus or whether a sufficient protection would ensue when administered via various routes or at different age groups. Nanocarrier/NP-based delivery systems can generally protect nanovaccines from premature degradation, increase stability, have excellent adjuvant properties, and may help in the targeted/controlled delivery of immunogens to antigen-presenting cells (APCs).

Incorporation of antigens in various NPs may be achieved via conjugation (covalent modifications) and/or by encapsulation (physical entrapment); these NPs incorporating antigens could exert local depot effects for ensuring the presentation of a specific antigen to immune cells [111]. Generally, the probability of drug encapsulation, modifications by polymers (e.g., polyethylene glycol (PEG), carbohydrates, among others), or modular functionalization by the fabrication of stable structures could all lead to improved drug delivery and optimized drug dosing via enhanced stability and drug retention times [112,113,114,115,116]. Immune cells generally express diverse surface receptors, namely the scavenger receptor, toll-like receptor, and mannose receptor [117]. Modifying the NPs/nanocarriers’ surfaces with a diversity of directing moieties (e.g., antibodies) allows the transport of viral antigens directly to particular surface receptors, thus inciting specific and selective mucosal or robust immune reactions. Indeed, NPs coated with immune cell-targeting molecules, such as antibodies, peptides, and carbohydrates [118,119,120], can be targeted with these overexpressed receptors to increase the adjuvant delivery and antigen efficacy for the promotion of a specific and selective or robust immune response in prophylactic nanovaccines.

#### CoVs and Nanovaccines

Vaccination is generally the most cost-effective way and affordable strategy to prevent, control, and fight against infections, especially those leading to several respiratory or pulmonary diseases. To date, vaccine formulations include subunit protein antigens, live-attenuated viruses, or inactivated/killed pathogens, which can elicit an antigen-specific immune response. Conventionally, live-attenuated vaccines present a reversion risk to their pathogenic virulence under a certain immunocompromised condition, whereas inactivated vaccines mostly lead to weak immune responses. Some vaccines based on protein subunits have also been developed to overcome these problems. The formulations of these vaccines can suffer from a reduced immunogenicity, and the protection induced is largely partial. In response to these risks, it is immensely crucial to develop risk-free and effective new vaccines in conjunction with nanotechnology-driven drug delivery systems, an essential requirement to achieve desired cell-mediated immunity against specific infections. Recent vaccine development efforts have mainly focused on the CoV transmembrane spike (S) glycoprotein, which extends from the viral surface and mediates host cell entry [121]. SARS-CoV-2 S requires angiotensin-converting enzyme 2 (ACE2) to pass into cells. The receptor-binding areas of SARS-CoV S and SARS-CoV-2 S attach with similar affinities to human ACE2, thus causing the effective spread of SARS-CoV-2 in large human populations. SARS-CoV-2 S glycoprotein shelters a furin cleavage site at the margin of S1/S2 subunits, which distinguishes this virus from SARS-related CoVs and SARS-CoV. Additionally, SARS-CoV-2 S ectodomain trimer was chosen to provide a blueprint for designing vaccines and inhibitors of viral entrance. SARS-CoV S murine polyclonal antibodies effectively obstructed SARS-CoV-2 S mediated entrance in cells [122].

It has been shown that the vaccination of mice with CoV S NPs could produce remarkable amounts of neutralizing antibodies against the homologous virus, although cross-protection against the heterologous virus was not provided [123]. It was suggested that CoV S NPs are capable of provoking precise anti-MERS- and SARS-CoV antibody responses in mice; however, this does not provide the overall anti-CoV response for protection against multiple CoVs, and further studies should be undertaken regarding nanovaccines for these purposes. Some adjuvants can be employed to accelerate the generation of neutralizing antibodies to CoV S. For example, alum and matrix M1 were employed with a remarkable increase in neutralizing antibodies (Alum: 15 fold and Matrix M1: 68 fold) over the neutralizing antibody levels generated from S NPs alone. Alum is considered as typical adjuvant employed to increase the neutralizing antibodies for SARS-CoV in a VLP vaccine approach [124]; additionally, it remarkably increased responses to MERS-CoV S NPs and SARS-CoV S NPs in mice [123].

Innovative studies and further investigations are required to validate if CoV S NP vaccinations can block CoV pathogenesis and replication in vivo, and subsequent further evaluations could be undertaken in animal models [125]. In neutralization evaluations, low-level, non-specific neutralization activity of PBS-vaccinated mice was observed, although all adjuvanted MERS-CoV S NP vaccinated mice showed remarkably elevated corresponding activities. Additionally, Matrix-M1 adjuvant increased the anti-MERS-CoV S neutralizing antibody reaction in vaccinated mice. It was revealed that mice vaccinated with MERS-CoV S NPs with Matrix-M1 were capable of proficiently and entirely blocking MERS-CoV replication in lungs; the Matrix-M1 NP with MERS-CoV S was a major candidate in terms of additional advancements for applications in humans or camels [125].

In one study, two kinds of vaccines for MERS-CoV were investigated, which included spike protein NPs prepared with alum adjuvant and recombinant adenovirus serotype 5 encoding the MERS-CoV spike gene (Ad5/MERS) [87]. These vaccines stimulated precise immunoglobulin G against MERS-CoV, although neutralizing antibodies against MERS-CoV were stimulated solely by homologous and heterologous prime-boost immunizations with spike protein NPs. Remarkably, the activation of Th1 cells was stimulated by immunization programs containing Ad5/MERS, but not by those containing spike protein NPs alone [87]. Heterologous prime-boost vaccination schedules comprising Ad5/MERS provoked concurrent Th1 and Th2 reactions, while homologous prime-boost schedules did not. Therefore, heterologous prime-boost might stimulate prolonged immune reactions against MERS-CoV due to a proper balance of Th1/Th2 responses [87]. However, both of these vaccination approaches could offer defense from MERS-CoV challenge in mice; a heterologous approach by priming with Ad5/MERS and boosting with spike protein NPs may be a promising and effective prophylactic approach against MERS-CoV infection [87].

In one investigation, by expressing the structural proteins of MERS-CoV in silkworm larvae and Bm5 cells, some vaccine candidates were prepared. Consequently, the spike S protein of MERS-CoV came up short on its transmembrane, and the cytoplasmic areas (SΔTM) were discharged into the hemolymph of silkworm larvae utilizing a bombyxin signal peptide and refined by applying affinity chromatography. The purified SΔTM generated small NPs as well as the full-length S protein and displayed the capability of binding to human dipeptidyl peptidase 4 (a receptor of MERS-CoV) [126], thus revealing that bioactive SΔTM could express in silkworm larvae. For the production of MERS-CoV-like particles, the co-expression of spike proteins has been completed in Bm5 cells, and envelope (E) and membrane (M) proteins secreted E and M proteins extracellularly, signifying that MERS-CoV-LPs might be generated. Although S protein has not been detected on virus-like particles (VLPs), E and M proteins have been located in the culture supernatant; S protein-displaying nanovesicles (~100–200 nm) were formed and confirmed by immuno-transmission electron microscopy (TEM). These purified SΔTM can be deployed to produce innovative NP-based vaccines against CoVs and in diagnostic detecting approaches [126].

In the process of vaccination, the utilizing of vaccine adjuvants and follow-up evaluations are very critical. For instance, silver NPs can be deployed as an adjuvant in mucosal vaccines based on inactivated influenza virus, which stimulates the remarkable antigen specific IgA formation with low toxicity by stimulating bronchus-associated lymphoid tissue (BALT) neogenesis [127]. Additionally, in one study, gold NPs and toll-like receptor agonists (applied as operative adjuvant in UV-inactivated SARS–CoV vaccine) were investigated as vaccine adjuvants for application with recombinant S protein [50]. Consequently, the immunization of mice was accomplished with more than 0.5 µg S protein without escape of adjuvant from SARS after infection with mouse-adapted SARS–CoV; however, eosinophilic infiltrations were detected in the lungs of the mice with immunization [50]. It was observed that gold NP-adjuvanted protein stimulated remarkable response of IgG but was unsuccessful in improving the efficiency of vaccine or reducing eosinophilic infiltration, due to significantly allergic inflammatory responses. While comparable virus titers have been detected in control animals and animals immunized with S protein with or without gold NPs, Type 1 interferon and pro-inflammatory replies were reasonable in the mice treated with S protein with and without gold NPs. Additionally, the Toll-like receptor agonist-adjuvanted vaccine triggered remarkable protective antibodies deprived of eosinophilic infiltrations, in addition to responses of Th1/17 cytokine [50].

### 2.3. QDs and GO against CoVs Infections

**Figure 8:**
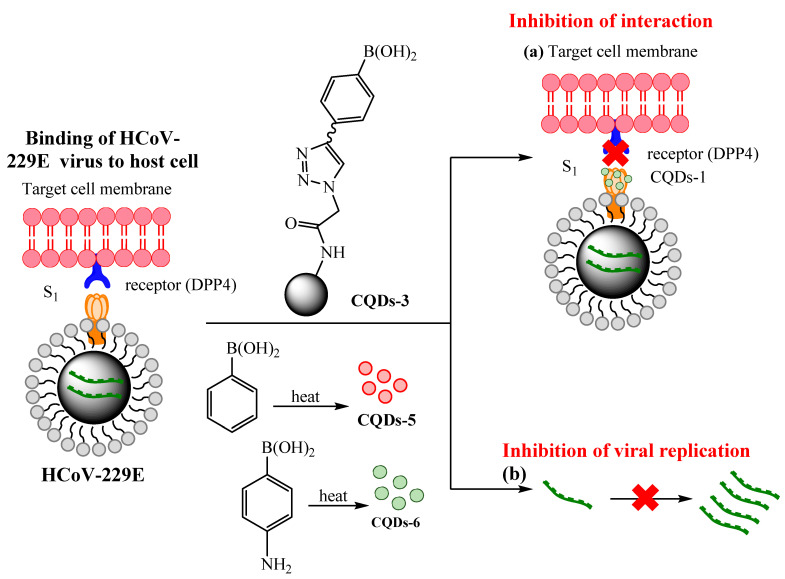
Carbon quantum dots (CQDs) prepared using hydrothermal carbonization and their influence on binding of HCoV-229E virus to cells: (**a**) inhibition of protein S receptor interface, (**b**) inhibition of viral RNA genome replication. Redrawn with permission from [57]. Copyright ^© 2020^, American Chemical Society.

QDs can be employed as perfect alternatives against pathogenic human CoVs infections; it is a very challenging issue, because these viruses vary biologically and can quickly change. In one investigation, the antiviral activities of seven different carbon quantum dots (CQDs) for treating human CoV HCoV-229E contagions were evaluated. The CQDs, about 10 nm in size with remarkable solubility in water, were synthesized via hydrothermal carbonization of carbon precursors, citric acid/ethylenediamine, and post-synthetic modification deploying boronic acids; concentration-dependent virus inactivation (EC50 of 52 ± 8 μg mL^−1^) was discernible from these ensuing nanomaterials [57]. Additionally, CQDs were also prepared from 4-aminophenylboronic acid with no additional modifications with much lowered EC50 of 5.2 ± 0.7 μg mL^−1^. Suggested mechanisms revealed that the HCoV-229E entrance inhibition is possibly due to the interaction of the functional groups of the CQDs with entry receptors of HCoV-229E; additionally, the viral replication step also displayed similarly big inhibition activity (Figure 8) [57].

**Figure 9:**
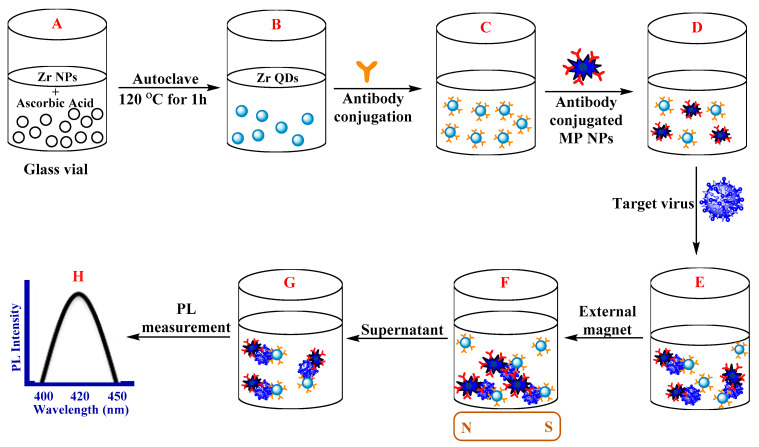
Biosensor design representation: (**A**) zirconium (Zr) NPs and CoV under reducing agent; (**B**) Zr QDs preparation; (**C**) the Ab linked QDs; (**D**) adding Ab-conjugated magneto-plasmonic (MP) NPs; (**E**) the generation of nanostructured MP fluorescent with the target’s addition and subsequent separation process (**F**); (**G**) dispersion of the nanohybrid-conjugated part and the following measurement of the optical characteristics (PL: photoluminescence) (**H**). Redrawn with permission from [53].

For detection of CoVs, some innovative approaches have focused on application of QDs. As an example, chiral zirconium QDs were prepared using *L*(+)-ascorbic acid, which showed fluorescence and circular dichroism characteristics [53]. The cytotoxicity of the prepared QDs was evaluated against rat brain glioma C6 cells using 3-(4,5-dimethylthiazol-2-yl)-2,5-diphenyltetrazolium bromide (MTT) assay. The anti-infectious bronchitis virus (IBV) antibodies of CoV were linked to these QDs to produce an immuno-link at the existence of anti-IBV antibody-conjugated magneto-plasmonic NPs and the target analyte. The fluorescence characteristics of the nanohybrids (immuno-conjugated QD-magneto-plasmonic NPs) via the external magnetic field-prompted partition process provided biosensing of CoVs (detection limit being: 79.15 EID/50 μL) (Figure 9) [53].

The wide-spectrum antiviral activities of graphene oxide (GO) against the porcine epidemic diarrhea virus (PEDV, an RNA virus) and pseudorabies virus (PRV, a DNA virus) have been evaluated; GO remarkably inhibited the infection of these viruses, at non-cytotoxic concentrations, for a 2 log diminution in virus titers [128]. Importantly, GO showed effective antiviral activities when linked with PVP, a nonionic oligomer, however not when linked with a cationic polymer, poly (diallyldimethylammonium chloride) (PDDA). Moreover, graphite and GO demonstrated poorer antiviral activity than monolayer GO and reduced graphene oxide (RGO), signifying that the nano-sheet constitution is crucial for antiviral characteristics. Importantly, GO incapacitated the viruses by structural damage before the virus could enter into cells [128]. In another investigation, Ag NP-modified GO nanocomposites, self-accumulated via electrostatic energy, were employed to inhibit entry into host cells for respiratory syndrome and porcine reproductive virus (~59% inhibition). Additionally, this nanocomposite prescription obstructed virus proliferation by accelerating the formation of IFN-stimulating genes (ISGs) and interferon-α (IFN-α) [129]. It appears that these nanomaterials can be considered as promising antiviral agents and should be further investigated for CoVs, especially SARS-CoV-2.

## 3. Current Challenges and Future Prospects

The incorporation of nanoscience for infectious disease treatment offers novel opportunities to overcome existing challenges of conventional drug therapies and presents therefore massive potential towards the enhanced mechanisms of action of recently accessible pharmaceuticals or the advancement of novel nano-therapeutics and diagnostics that are urgently needed in the drug resistance age. NPs/nanocarriers or nanovaccines (especially nanostructures functionalized with proper functional groups) could be effectively designed to integrate traditional antiviral features with those amendments. Such alterations are distinctive of advanced nanosystems with a high/large surface-volume ratio, ultra-small size, and the potential abilities for tailoring the surface with the multi-functionalization probability. Thus, they could be applied to interface with numerous viruses along with preventing their entrance into cells/tissues.

Generally, from the management point to the site of antiviral activities, pharmaceuticals may encounter a diversity of biological barriers [130,131,132]. One main instance is the setting up of latent reservoirs in cellular and structurally immune-privileged sites, for example, the blood-brain or blood-testis barrier. Therapeutic size is a pivotal consideration in successful drug development for transmissible diseases, where entrance to unreachable compartments, namely viruses crossing the blood-brain barrier, is obligatory for preventing the creation of dormant infections with persistent replication at low levels [133]. Nanoparticulate drug transporters are generally proficient at crossing such membranes and are thus a favorable means to be studied for avoiding this impediment. The utilization of NPs or nanocarriers to ease entrance into diverse subcellular sections is consequently an outstanding choice because of their small size, architecture, or specific properties; they have been therefore developed for viral detection or treatment and are adequately capable of surmounting natural obstructions to attain specific and targeted delivery [9].

Nanomaterials have recently appeared to exert their antiviral activities against viruses by different mechanisms, making them attractive tools for viral treatment. All immunological assets and benefits of nanostructures can ensue from the combination of the following features: (i) surface charge tenability (that could assist cellular entrance/export through the cell membrane) [134], (ii) small particle size (to enable drug transfer into immune-favored locations) [21,112], (iii) large surface area (to ensure that they can accommodate the large drug payloads) [113], and (iv) intrinsic antiviral assets (that can display biomimetic properties) [135,136], for example, dendrimers and silver NPs [137,138].

Besides these, nanostructures possess other exceptional properties, namely their capability to deliver viral antigens in a controlled manner, their capacities to mimic the viruses in terms of structure or size without requiring a real infection, and their capabilities to activate follicular dendritic cells or B cells, antigen cross-presentation, as well as induce a humoral/cellular immune response. Collectively, the clinical applications of NP-based therapeutics are revolutionizing several medical fields, namely diagnosis, treatment, control/management, prevention, and detection, such as nanocarriers for viral infections and delivery of nanodrugs or bioactive compounds. Consequently, nanodrug delivery can be immensely improved by these nanostructures with the desired directing entities to enhance specificities for preferred target tissues, cell types, or subcellular compartments [134,139]. Indeed, some approved nanodrug delivery systems and nanovaccines are making an actual revolution in viral disease prevention, treatment, and management; nanotechnology offers new prospects towards the manufacturing of vaccine nanoformulations [140,141].

NP-based vaccines (or nanovaccines) are envisaged to be more effective, safe, economic, or convenient than subunit and conventional vaccines. Nanovaccines may provide an excellent future ahead if the following goals are attained: (i) stabilization at ambient temperature via encapsulation of vaccines in NPs, (ii) exploration of replaceable/interchangeable routes of administration, and (iii) facilitation of controlled/specific release at a particular location. They may be reinforced once, unlike conventional vaccines which are generally required to be reinforced many times. Indeed, nanotechnology platforms afford a new choice and a promising opportunity to improve cellular or humoral immune responses; nanovaccines may be induced either by cellular immunity (cytotoxic or helper T-cell responses) or humoral immunity (B-cell responses). The advantages are ascribable to the nanoscale particle size that can facilitate uptake via phagocytic cells and the mucosa/gut-associated lymphoid tissues, leading to efficient antigen processing and recognition.

One of the probable methodologies could be based on the utilization of NP-based vaccination [142]. Nanotechnology presents diverse benefits in the areas of detection, management, and treatment, either via site-specific drug delivery or targeted delivery of particular medicines in a controlled manner. Nanoscience has been recently applied to improve and develop vaccine efficacy as well as optimization utilizing several advanced nanosystems to improve antigen delivery and efficient induction of humoral/cellular immunity [143,144]. One of the most crucial advantages of using NPs in vaccine systems is that they could deliver proteins and peptides to specific tissue cells and help them to produce a robust immune response [144,145]. Indeed, these NPs can protect the applied antigens from premature proteolytic or enzymatic degradation, control release, simplify antigen uptake, and are safe for human consumption. For some viruses, including SARS-CoVs, RSV, and acquired immunodeficiency syndrome (HIV/AIDS), no effective vaccines are presently accessible making infection detection and treatment a critical necessity. Today, three viruses, namely MERS- and SARS-CoVs, and SARS-CoV-2, present key ultimatums for management since there are still no specific anti-CoV drugs accessible. Owing to the involvement of COVID-19, many facts regarding virulence, pathogenesis, and the real source of viral infection or mode of transmission still need to be addressed; some suggestions and potential alternatives for further evaluations, especially for SARS-CoV-2, include:

- The effects of metallic NPs (e.g., silver, gold and copper NPs or hybrid nanomaterials) can be evaluated against SARS-CoV-2 [17]. For instance, silver NPs showed inhibition abilities against the viral entrance in host cells, for HIV-1 virus, as these NPs are capable of interacting with the cell receptors [146]. Additionally, gold NPs stabilized by biocompatible polymers demonstrated antiviral activity against HIV-1 and some subtypes of influenza virus (e.g., H1N1, H3N2, H5N1) [17,146].
- QDs, metal-grafted GO, nanocomposites (e.g., CuO nanocomposites), and nano-phytotherapeutics can be considered for their potential antiviral evaluations [17,147].
- Biodegradable nanocarriers and carbon nanotubes can be proposed as potential antiviral agent carriers. For instance, antiviral drugs such as ribavirin and isoprinosine have been chemically linked on a single-walled carbon nanotube surface to transmit these antiviral compounds across biological membranes [148].
- Multi-functionalization should be performed for drug delivery platforms, imaging, and detection systems for viral localization and specific tissue or organ targeting. Development of toxicity and the reduction of side effects as well as bioavailability of antiviral drugs can be further explored based on nanotechnological insights; nanotrap particles showed suitable inhibition effects against influenza viruses, which can be explored for other viruses [149].
- Importantly, multidrug NPs (with high biocompatibility and drug loading) for the mitigation of uncontrolled inflammation is very promising, especially in the case of COVID-19. In one study, NPs were produced by conjugating squalene, an endogenous lipid, with adenosine, an endogenous immuno-modulator, and then encapsulating α-tocopherol, a natural antioxidant [150]. By using the vascular endothelial barrier dysfunction at sites of acute inflammation, these multidrug NPs could transport the therapeutic agents in a targeted manner and confer remarkable survival advantages to the treated animals in lethal models of endotoxemia. The selective delivery of adenosine and antioxidants together could serve as an innovative method for the treatment of acute inflammation with reduced side effects and significant therapeutic potential, especially in the case of cytokine storm (hypercytokinaemia) ensued by COVID-19 [150].
- Rapid and reliable tests for the new CoV are critical for controlling the pandemic, such as innovative biosensors designed for SARS-CoV-2 detection by applying nano-based technology; the aforementioned biosensor designed by Qui et al. [70] is an example of such.

## 4. Conclusions

Nanomaterials can be considered as perfect candidates against viral infections, especially CoVs, because of their ability to enter cells easily and interact with viruses and avoid viral genome replication. NPs can be applied as a measure to revert antiviral resistance, which is a gradually developing challenge of conventional therapeutics presently available. Furthermore, improvements, especially enhancing the bioavailability and reducing the toxicity of conventional antiviral drugs, can be executed by using groundbreaking advancements in nanotechnology. Nowadays, researchers are focusing on high-level experimental studies to find innovative and smart nano/bio-materials and matrices for designing controlled release and targeted drug delivery systems; nanovaccines and nano/bio-sensors against pathogenic viruses, particularly for human and animal CoVs, need more exploration. In the history of mankind, different cases of epidemics and pandemics have claimed the lives of many people. In the meantime, however, power to control a crisis, innovative solutions, intelligent management, and the use of smart and modern technologies, especially exploiting the field of emerging nanotechnology, could be very effective in managing the current and future outbreaks.

## Figures and Tables

**Table 1:** Summary of developed nanoparticulate drug delivery systems towards treatment and/or therapy of viral infections.

Nanoplatform		Properties (Shape/Size/Toxicity)	Drug	Virus	Refs.
**NPs**	Silver (Ag)	Monodisperse and uniformly spherical; highly stable Ag@AM (2 nm > 28 days); Ag NPs loaded with AM on their surface less cytotoxic (~90%) than free AgNPs (65%) or AM (56%)	Amantadine (AM)	H1N1	[30]
	Selenium (Se)	Uniformly spherical Se@AM (70 nm)—more stable, superior antiviral effect on kidney cells treated with H1N1 and less cytotoxicity (79.26% viability) than SeNPs (200 nm, 58.8%) and/or free AM (53.23%)	AM		[31]
		Uniformly spherical Se@OTV (100 nm)—superior antiviral effect on kidney cells treated with H1N1 and less cytotoxicity (93% viability) than SeNPs (60%) and/or free OTV (53%)	Oseltamivir (OTV)		[32]
	PEG-PLGA	Uniformly spherical shape; NPs (178 nm & 197 nm) loaded with diphyllin and bafilomycin; high biocompatibility and antiviral activity towards the NP drugs than the free drugs	Diphyllin & Bafilomycin		[33]
	PLGA	Uniform, spherical/smooth surface NPs (116−143 nm); three GCV pro-drugs can be separately loaded on PLGA NPs; non-cytotoxic PLGA NPs (24 h and 48 h contact of three diverse NPs concentrations with HCEC cell)	Gangiclovir (GCV)	HSV ^a^ -1	[34]
	HPAC	99% drug loading efficiency; HPAC (diverse concentrations)—non-cytotoxic towards human epithelial cells (vaginal, corneal), foreskin fibroblasts, HeLa cells: cell viability >75%	Acyclovir (ACV)	HSV	[35]
	PLGA	Polydisperse particles loaded with LAM (221−250 nm); slow NPs degradation in simulated intestinal fluid PBS; the molecular interaction between polymer and LAM confirmed by FTIR and DSC	Lamivudine (LAM)	HIV	[36]
	PLGA	Spherical/smooth surface NFV NPs (~185 nm); almost narrow distribution	Efavirenz (EFV)		[37]
	PEO-PCL	Spherical PEO-PCL NPs (~200−270 nm)with smooth surface; SQV is encapsulated into NPs	Saquinavir (SQV)		[38]
	PLGA-PEG	Spherical shape (~125 nm) for those loaded with SAHA and NFV; ~119 nm for those loaded with SAHA; ~118 nm for NPs loaded with NFV	Suberoylanilide hydroxamic acid (SAHA) and NFV		[39]
	Hybrid NPs (PLGA, PLA, MMA-SPM, and PMMA)	Almost spherical NPs; PLGA NPs (58~224 nm) and MMA-SPM NPs (91−823 nm); nontoxic NPs (male mice)	LAM+AZT (Zidovudine)		[40]
	PMA coated MNP	Uniformly spherical NPs conjugated with ENF (35.2 nm); nontoxic in vivo and in vitro NPs conjugated with ENF	Enfuvirtide (ENF)		[41]
**Nanospheres (NS)**	Chitosan (Cs)	Spherical NS (~200 nm diameter) with smooth surface; polydisperse NS; after contact with NS has a satisfactory Vero cell viability; 86% ACV encapsulation efficiency	ACV	HSV	[42]
**Dendrimers**	PG ^b^	Antiviral effect in vitro; nontoxic peptide-PG conjugates in vitro	Peptides	IAV ^c^	[43]
**Lipid NPs (LNPs)**	DSPC ^d^ +MPEG+DSPE	LNPs (52~68 nm); in vivo antiHIV LNPS do not display local reactions, and also animal platelet counts were within normal limits	LPV ^e^ +TFV ^f^ +RTV ^g^	HIV	[44]
	PEG and phospholipids	LNPs loaded with drug or drugs (33~68 nm); incorporation efficiency (88~96%)	ATV ^h^ +TFV+RTV		[45]
**Micelles**	Cs-*g*-oligo(NiPAam)	Copolymers self-assembled in multimicellar aggregates by hydrodynamic (330~436 nm); high mucoadhesion and cytocompatibility properties	EFV	HIV	[46]

^a^ Herpes simplex virus; ^b^ polyglycerol; ^c^ influenza A virus; ^d^ 1,2-Distearoyl-sn-glycero-3-phosphocholine; ^e^ Lopinavir (LPV); ^f^ Tenofovir; ^g^ Ritonavir; ^h^ Atazanavir.

**Table 2:** Engineered nanomaterials to detect and inhibit CoVs.

NPs	Virus	Mechanistic Aspects	Purpose	Refs.
Chiral gold NPs-quantum dot (QD) nanocomposites	CoVs	Chiral plasmon–exciton systems	Viral detection	[47]
Graphene oxide (GO) sheets	Feline CoVs	Organism models: fcwf-4 cells, DF-1 cells; association with viral lipid tails leading to aggregation and rupture of the envelop	Viral inhibition	[48]
GO sheets with silver particles	Feline CoVs	Association with viral lipid tails leading to aggregation with attachment of silver NPs with –SH group of protein and rupture of the envelop	Viral inhibition	[48]
Carbon electrodes modified with gold NPs	MERS CoVs	Recombinant spike protein S1 has been employed as a biomarker; the sensor is based on indirect competition between free virus in the sample and immobilized MERS-CoV protein for a fixed concentration of added antibody to the sample	Viral detection	[49]
Gold NP–adjuvanted S protein	SARS-CoVs	Stimulates significant antigen–specific IgG response against SARS–related CoV infection	Development of vaccines against severe pneumonia–associated CoVs	[50]
Cationic carbon dots based on curcumin	CoVs; porcine epidemic diarrhea virus (PEDV)	Inhibition of the viral proliferation; the structure of surface protein in viruses has been changed, thus prohibiting viral entry; cationic carbon dots based on curcumin can suppress the synthesis of negative-strand RNA and budding of the virus, and the accumulation of reactive oxygen species by virus. Further, it can suppress viral replication by stimulating the formation of interferon-stimulating genes (ISGs) and pro-inflammatory cytokines	Antiviral properties	[51]
Glutathione-capped Ag_2_S nanoclusters (NCs)	PEDV as a model of CoV	Ag_2_S NCs treatment prohibited the formation of viral negative-strand RNA and viral budding. It positively regulated the production of IFN-stimulating genes (ISGs) and the expression of pro-inflammation cytokines, which may inhibit the virus infection; inhibition of CoV proliferation	Viral inhibition	[52]
Chiral zirconium QDs	CoVs	The fluorescence properties of immuno-conjugated QD-magneto-plasmonic NPs	Viral detection	[53]

**Table 3:** Some important Au NP-based strategies towards the detection of viruses [69].

Virus	Target	Au NP Systems		Assay	Detection Limit & Detection Range	Time (min)
		Shape & Size (nm)	Biomolecule			
SARS- CoV	NC ^a^ protein	Spherical, 70	DNA	Electrochemical	2.5 pM, 50–2.5 pM	>120
	PP1ab ^b^ gene	Spherical, 13	None	Colorimetric	60 fmol, ND	5
MERS-CoV	E protein gene (upE) and open reading frames (ORF) 1a	Spherical, 19	DNA	Colorimetric	1 pmol/μL	10
HCV	HCV antigen	Spherical, 12	DNA	Electrical	1 pg/μL, 10 ng/μL−1 pg/μL	245
	HCV Ab	Spherical, 15	SPA ^c^	Scanometric	3 ng/mL, 3 μg/mL−3 ng/mL	10
	Core gene	Spherical, 8~15	DNA	DLS/Colorimetric	0.36 pM, 0.3 μM−0.3 pM	75
	Full genome	Spherical, 15	None	Colorimetric	50 copies, ND	110
		Spherical, 8	DNA	Fluorometric	300 fM, 550−15 pM	184
	5′ UTR ^d^ gene	Spherical, 10~15	DNA	Electrochemical	~1 pM, 2.0−0.01 nM	60
		Spherical, 40		Colorimetric	2 fmol, 30−2 fmol	50
HBV	Anti-HBV	Spherical, 15	SPA	Scanometric	3 ng/mL, 3 μg/mL−3 ng/mL	10
	C ^e^ gene	Spherical, 10	DNA	Scanometric	1 fM, 10^−11^−10^−15^	90
	S ^f^ gene	Spherical, 8~15	DNA	Colorimetric	0.36 pM, 0.3 μM−0.3 pM	75
		Spherical, 5	Avidin	Electrochemical	0.7 ng/mL, 1.47−0.7 ng/mL	105
		Spherical, 13	DNA	Scanometric	20 fM, At 20 fM	330
	HBV DNA	Rod, ND	DNA	Fluorometric	15 pM, 6.0−0.045 nM	~60
	HBeAg ^g^	Rod, L = 46, D = 13	Ab	Fluorometric	8.3 ng/mL, Up to 264 ng/mL	ND
	HBsAg	Spherical, 10, 50, 100	Ab	DLS ^h^	0.005 IU/mL, 1−0.005 IU/mL	˂60
		Spherical, 16	Ab	Electrochemical	0.1 ng/mL, 650−0.5 ng/mL	65
		Spherical, ~10	Ab		2.3 pg/mL, 1.0−0.01 ng/mL	60
		Spherical, 16	Ab		87 pg/mL, 1500−0.1 ng/mL	~50
		Rod, L = 46, D = 13	Ab	Fluorometric	9 ng/mL, Up to 288 ng/mL	ND
		Rod, L = 68, D = n30	Ab	LSPR ^i^	0.01 IU/mL, 1−0.01 IU/mL	ND
		Spherical, 5.5	Peptide	Fluorometric	0.1 pg/mL, 0.1−0.0001 ng/mL	30
H1N1	Anti-H1N1	Spherical, 20	Ab	DLS	˂100 TCID50/mL, 1.4 × 10^6^−5.5 × 10^3^ TCID50/mL	30
	NA gene	Spherical, ND	DNA	SERS ^j^	25 nM, 50−25 nM	ND
				Fluorometric		
	HA antigen	Spherical, 25	Protein A	Fluorometric	13.9 pg/mL, 800−12.5 ng/mL	ND
	M ^k^ gene	Spherical, ND	Avidin	Electrochemical	577 pM, 3−0.001 pmol	80–50
H5N1	NA ^l^ gene	Spherical, 15	DNA	Scanometric	100 fM &103 TCID50, ND	150
	HA ^m^ gene	Spherical, 32	Ab	Colorimetric	40–0.1 ng, 100−0.1 ng	ND
		Spherical, ND	DNA	Electrochemical	0.4 pM, 1.0 nM−5.0 pM	20
		Spherical, 3	Daunorubicin	Scanometric	10 pM, 10 pM−100 nM	20
		Spherical, 1.4	None		10 pM, 10 pM−100 nM	90
		Spherical, 15	DNA		100 fM & 103 TCID50	150
	Particles	Spherical, 10	Pentabody	Colorimetric	10 ng/mL, 10^−1^−10^−4^ μg/mL	35
		Spherical, 22	Ab	Fluorometric	0.09 ng/mL, 12−0.27 ng/mL	30
HSV-2	Anti-HSV-2	Spherical, ND	Ab	Colorimetric	ND, ND	20
HIV	P24 Antigen ^n^	Spherical, 15	Avidin/DNA	Scanometric	0.1 pg/mL, 500−0.1 pg/mL	~360
		Spherical, 30	Avidin/DNA	DLS	0.2 pM, 31.4 pM−1.6 fM	>170
		Spherical, 30	Ab/DNA	IPCR^o^	0.1 pg/mL, 1000−0.1 pg/mL	>85
		Spherical, 30	Ab/DNA		1 pg/mL, 10,000−1 pg/mL	>125
	*Pol*^p^ gene	Spherical, 13	DNA	DLS	10 fM, 10 nM−1 fM	80
		Spherical, 3	DNA	Electrochemical	0.34 fM, 1.0 µM−0.1 pM	50

^a^ Nucleocapsid; ^b^ ployprotein1ab; ^c^ staphylococcal protein A; ^d^ five-prime untranslated region; ^e^ core; ^f^ surface; ^g^ hepatitis B antigen; ^h^ dynamic light scattering; ^i^ localized surface plasmon resonance; ^j^ surface enhanced Raman scattering; ^k^ matrix; ^l^ neuraminidase; ^m^ hemagglutinin; ^n^ P24 capsid protein antigen; ^o^ immuno-PCR; ^p^ polymerase gene; gene.

**Table 4:** Some important examples of NP-based vaccine candidates against CoVs.

Type of CoVs	NPs	Adjuvant	Findings	Refs.
Swine TGEV	Gold NPs	Gold NPs	Acceleration of the peritoneal macrophages respiratory activity and plasma IFN-γ level	[84]
SARS-CoV	Gold NPs	Gold NPs	Stimulation of IgG response	[50]
MERS-CoV (RBD antigen)	Ferritin-based NPs	-	Stimulation of CD^4+^ T-cells and IFN-γ TNF-α responses	[85]
MERS-CoV (RBD antigen)	Hollow polymeric NPs	STING agonist (cdGMP)	Stimulation of remarkable levels of humoral responses and IgG2a antibodies, with no stimulation in lung eosinophilic immunopathology	[86]
MERS-CoV	Spike protein NPs	Aluminum	Stimulation of significant titers of neutralizing antibody and Th2 immune response, with no stimulation of Th1 immune response	[87]

**Table 5:** Summary of diverse types of nanocarrier/NP-based vaccine delivery systems against viral infections.

Nanocarriers Delivery System	Virus	Antigen/Adjuvant	Disease	Route of Administration	Refs.
Gold NPs	HIV-1	Viral plasmid DNA	HIV/AIDS pandemic	Intradermal	[90]
Gold NPs	H1N1	Membrane matrix protein 2 (M2e)/CpG	Influenza	Intranasal	[91]
Gold NPs	FMDV ^a^	Viral protein	Foot and mouth disease	Intradermal	[92]
Gold NPs	H1N1, H3N2, H5N1	M2e/CpG	Influenza	Intranasal	[93]
Chitosan NPs	HBV ^b^	HBsAg	Hepatitis B	Intraperitoneal	[94]
Carbon magnetic NPs	-	Hen egg lysozyme	-	Intravenous	[95]
Chitosan NPs	NV ^c^	Live virus vaccine	Newcastle disease	Intranasal or oral	[96]
PCL ^d^/chitosan NPs	HBV	HBsAg	Hepatitis B	Intranasal	[97]
γ-PGA NPs	H1N1	Hemagglutinin (HA)	Influenza	Intranasal	[98]
VLPs	NV	Capsid protein	Norwalk virus infection	Oral	[88]
VLPs	HBV	Nucleocapsid protein	Hepatitis	Intravenous	[89]
VLPs	H3N2	Structural proteins, e.g., HA, neuraminidase, NA, and matrix (M1)	Influenza	Intramuscular	[99]
VLPs	RV ^e^ 8-2/6/7-VLP	Multiple proteins, e.g., cholera toxin (CT) and *Escherichia coli* heat-labile toxin (LT)	Rotavirus	Rectal	[100]
Polypeptide NPs	CoV	Viral protein (spike)	SARS-CoV	-	[101]
Alginate coated chitosan NPs	HBV	HBsAg	Hepatitis B	Intranasal	[102]
PLA and PLGA NPs	HBV	Hepatitis B surface antigen	Hepatitis B	Pulmonary or intramuscular	[103]
PLA and PLGA nano/micropraticles	TT ^f^	Tetanus toxoid	Tetanus	Intramuscular	[104]
PLGA NPs	BPI3V ^g^	BPI3V proteins	Bovine respiratory	Intranasal	[105]
Polyanhydride	RSV	F and G glycoproteins	Bovine respiratory syncytial	Intranasal	[106]
HPMA/NIPAM ^h^	RSV	F protein/TLR-7/8 agonist	RSV, influenza, HIV-1	Intramuscular, intranasal, intravenous	[107,108]
DLPC ^i^ liposomes	H1N1	M2, HA, NP/MPL ^j^ and trehalose 6,6′ dimycolate	Influenza	Intramuscular, intratracheal, intranasal	[109]
Cationic nanomicelles based on PSA ^k^	HIV-1	PSA/mRNA encoding	HIV/AIDS	-	[110]

^a^ Foot-and-mouth disease virus; ^b^ hepatitis B virus; ^c^ Newcastle virus; ^d^ poly-ε-caprolactone; ^e^ Rotavirus; ^f^ tetanus toxoid; ^g^ bovine parainfluenza 3 virus; ^h^ N-(2-hydroxypropyl)methacrylamide/N-isopropylacrylamide; ^i^ dilauroylphosphatidylcholine; ^j^ monophosphoryl lipid A; ^k^ polyethyleneimine-stearic acid.
